# Draft genome sequence of heat-tolerant *Escherichia coli* strain AKS1GF CIFRI isolated from a coal power plant discharge in the river ganga

**DOI:** 10.1128/mra.00953-24

**Published:** 2024-10-29

**Authors:** Amiya Kumar Sahoo, Dharmendra Kumar Meena, Basanta Kumar Das, Subhankhi Dasgupta, Smruti Samantaray, Debasmita Mohanty, Ayushman Gadnayak, Subhasmita Behera, Asit Kumar Bera

**Affiliations:** 1 ICAR-Central Inland Fisheries Research Institute, Barrackpore, Kolkata, India; DOE Joint Genome Institute, Berkeley, California, USA

**Keywords:** *Escherichia coli*, genome sequence, thermal discharge, river ganga

## Abstract

*Escherichia coli* strain, AKS1GF ICAR-CIFRI, was isolated from a thermally contaminated water with average water temperatures of 45°C ± 2.5°C in river Ganga, India. The draft genome sequence is of 4.7 MB consisting of 44 contigs. The strain contains 4,314 protein coding genes and 96 RNA genes.

## ANNOUNCEMENT

*Escherichia coli,* a widely distributed bacteria often found in the gut microbiota, has been shown to inhabit several ecological habitats, including aquatic environment (1). In order to examine the genetic foundation of *E. coli’s* ability to adapt at high-temperature habitat, we conducted a genome sequencing analysis of AKS1GF that was obtained from a thermal discharge site in the river Ganga, the largest river of India with cultural and ecological significance (2).

The *E. coli* strain AKS1GF was isolated from a soil sample collected from the thermal discharge site of a coal power plant in the river Ganga during April 2024 (87°53'39"E-87°56'41"E;24°40'19"N-24°52'12"N). The soil sample was serially diluted, and 10^−5^ dilution was transferred to Escherichia coli (EC) broth (Himedia, Mumbai, India) and incubated at 37°C for 24 hours. One loopfull of culture was transferred to MacConkey agar (Himedia, Mumbai, India) plate for isolation of pure culture. The single lactose-producing bacteria were picked from the MacConkey agar plate and transferred to Tryptone Soya Broth (TSB) for genomic DNA isolation. Genomic DNA was isolated using QIAamp DNA mini kit (Qiagen). A multiplex PCR was used to confirm the *E. coli* isolate by using five different primers (3), uidA (ß-D-galactosidase), lacZ (ß-D-galactosidase), lacY (lactose permease), cyd (cytochrome bd complex), and phoA (bacterial alkaline phosphatase).

Using the QIASeq FX DNA kit (Qiagen), a paired-end library containing the genomic DNA was created and used for sequencing on the Illumina NextSeq 2000 platform. A total of 5.5 Gb was generated (Table 1). To check the quality of the data, FASTQ files was pre-processed using FastQc v0.11.9 (4), following the specifications in MultiQC report ((5). To filter adapter contamination, the fastp tool (v0.12.4) (6) was used. The genome was assembled with Megahit (7). The genome was made up of 44 contigs and had a N50 value 239.8 kb, as indicated in Table 1. Annotation was performed using the NCBI Prokaryotic Genome Annotation Pipeline (8) and revealed 4,501 genes, with 4,405 coding sequences and 96 noncoding sequences (12 rRNAs, 74 tRNAs, and 10 noncoding RNAs).

**TABLE 1:** Overview of the draft whole-genome sequence of *E. coli* strain AKS1GF

Sequencing method	Illumina NextSeq 2000
No of reads (bp)	20,000,000 (paired-end)
Average length of the paired-end reads	119 bp
Genome size	4.7 mb
No. of contigs	44
N50 (bp)	239.8 kb
GC%	51
Genome coverage	50.7×
Protein coding genes	4,314
Completeness	99.52%
Contamination	0.44%
Biosample accession no	SAMN42748805

## Data Availability

The whole-genome sequence has been published in the NCBI DDBJ/EMBL/GenBank database under the accession number JBGDMG000000000. The version mentioned in this study is JBGDMG000000000.1, while the raw reads were submitted to SRA under run number SRR29979039.
